# Interlayer-confined redox in Ti₃C₂Tₓ-MXene laminates: a reagent-free screen-printed platform for the detection of erdafitinib

**DOI:** 10.1007/s00604-026-08175-4

**Published:** 2026-06-04

**Authors:** Bharat Prasad Sharma, Malik Wasim Abbas Chun, Marwan Shalash, Motee Lal Sharma, Abdulraheem SA Almalki, Mohamed H. Helal, Xiong Jinping, Selcan Karakuş

**Affiliations:** 1 College of Materials Science and Engineering, Beijing University of Chemical Technology, Beijing, 100029 PR China; 2 School of Cancer and Pharmaceutical Sciences, King’s College London, London, UK; 3 Department of Pharmaceutics and Pharmaceutical Technology, Faculty of Pharmacy, Zarqa University, Zarqa, 13110 Jordan; 4 Central Department of Chemistry, Tribhuvan University, Kathmandu, 44600 Nepal; 5 Department of Chemistry, College of Science, Taif University, P.O. Box 11099, Taif, 21944 Saudi Arabia; 6 Center for Scientific Research and Entrepreneurship, Northern Border University, Arar, 73213 Saudi Arabia; 7 Department of Chemistry, Faculty of Engineering, Istanbul University- Cerrahpaşa, Istanbul, 34320 Türkiye; 8 Health Biotechnology Joint Research and Application Center of Excellence, Esenler, Istanbul, 34220 Türkiye

**Keywords:** MXenes, FGFR inhibitors, Interlayer-confined redox, Resource-processing waters, Free-standing electrodes

## Abstract

Electrochemical MXene sensors are attractive for point-of-care analysis but are often limited by MXene oxidation, reliance on soluble mediators, and multi-step surface chemistries that complicate their use in complex samples. Herein, an interlayer-confined redox (ICR) strategy is implemented based on Ti_3_C_2_T_*x*_-MXene laminates, where Ti_3_C_2_T_*x*_ is co-filtered with methylene blue (MB) to trap a redox mediator network within its galleries. These free-standing laminates are readily cut and transferred to commercial screen-printed electrodes (SPEs) featuring carbon working and counter electrodes with an integrated Ag pseudo-reference, yielding a sharp, intrinsic voltammetric MB signal at − 0.25 V vs. on-chip Ag without the need for external reagents. Structural and spectroscopic analyses confirm gallery expansion, intact in-plane crystallinity, absence of crystalline TiO_2_, minimal mediator leaching, and robust stability through repeated cycling and storage. Optimized differential pulse voltammetry (DPV) with the ICR-MB-MX-11 laminate (optimized from ICR-MB-MX-*x* variants, *x* = 4, 7, 11, 14, based on filtrate MB concentration) enables reagent-free detection of the fibroblast growth factor receptor (FGFR) inhibitor erdafitinib (ERD) across 0.05–10.5 µM, achieving a 0.01 µM limit of detection (LOD), ≤ 0.8% intra-/inter-electrode relative standard deviation (RSD; *n* = 3), and 98 − 102% recoveries in spiked, PBS-diluted human urine. Density‑functional calculations indicate that ERD adsorption perturbs the MB‑induced electronic arrangement at the Fermi level, consistent with a mediator‑gated inhibition mechanism. More broadly, the reagent-free MXene/SPE configuration may also support field-deployable analysis of complex aqueous matrices relevant to environmental and resource-processing applications.

**Graphical Abstract:**

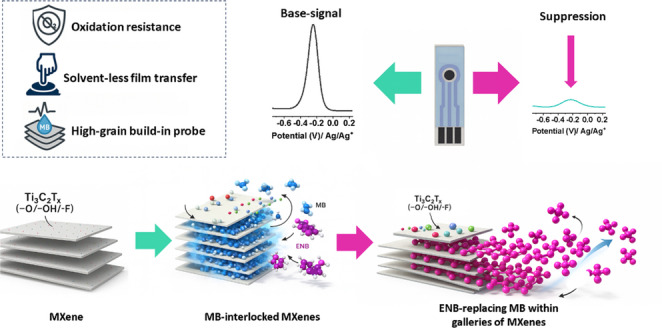

**Supplementary Information:**

The online version contains supplementary material available at 10.1007/s00604-026-08175-4.

## Introduction

Electrochemical sensors are steadily moving toward disposable, reagent-free formats that operate reliably in complex samples without the need for external labels or soluble mediators [1]. 2D MXenes, and Ti_3_C_2_T_*x*_ in particular, are compelling transducer materials because they combine metallic conductivity, hydrophilic terminations (–O, –OH, –F), and a high density of accessible surface sites that promote interfacial charge transfer and molecular adsorption, attributes that have underpinned a rapidly expanding catalog of chemical and biosensing platforms [2–4]. More broadly, MXenes bridge mineral-derived transition-metal chemistry and advanced electrochemical materials, as ore-derived metal resources can be converted through MAX-phase precursors into functional 2D carbide/nitride platforms. When processed as aqueous inks, few-layer Ti_3_C_2_T_*x*_ dispersions can be drop-cast or printed onto screen-printed electrodes (SPEs), enabling the fabrication of low-cost devices with minimal instrumentation [5–8]. SPEs are widely recognized as disposable, economical, and scalable electroanalytical platforms for point-of-use analysis, although their performance depends strongly on ink composition, printed-film quality, and the reproducibility of the modifier/electrode interface [9, 10]. Despite these advantages, transitioning these laboratory demonstrations into robust, fieldable sensors remains challenging because the same aqueous environment that simplifies ink preparation also accelerates hydrolysis-driven oxidation of Ti–C to TiO_2_, degrading the inherent chemical characteristics and undermining long-term reproducibility [11–13].

Mitigation strategies, such as blending antioxidants into dispersions or polymer encapsulation after deposition, offer partial relief but introduce new trade-offs [14]. Antioxidants can extend shelf life; however, they can complicate formulation and compromise batch-to-batch consistency [15]. At the same time, polymer overlayers can slow oxidation but also hinder interfacial kinetics and occlude active sites, thereby offsetting the electronic advantages of Ti_3_C_2_T_*x*_ [16]. In parallel, many MXene-based analytical schemes still rely on soluble mediators, enzymes, or multi-step surface chemistries that add user steps and may cause mediator leaching, particularly in biological matrices. For example, methylene blue (MB) has been used in MXene-based sensors as a redox-active signal probe in ratiometric electrochemical platforms, such as MB-functionalized Ti_3_C_2_T_*x*_*-*MXene/Pt composites for carbendazim detection and magnetic MXene/MB nanocomposites for SARS-CoV-2 spike protein detection [17, 18]. MB has also been incorporated into MXene-based aptasensing architectures, together with AuNPs or other nanocomponents, to amplify the electrochemical response [48]. Although these approaches improve signal output, MB is generally considered a surface-associated or composite-based mediator, leaving the redox probe exposed to the electrolyte and potentially susceptible to desorption or matrix-induced instability [19]. Moreover, conventional mediator-based electrochemical sensors often require the mediator to be added externally or immobilized through additional surface chemistry, increasing operational complexity and compromising compatibility with disposable point-of-care formats. Therefore, the key challenge is not only to introduce a redox-active probe into MXene but also to confine it, preserving electrochemical addressability while suppressing mediator loss. Collectively, these limitations require an architecture that avoids prolonged solvent exposure, locks redox functionality in the solid state, and interfaces cleanly with disposable SPEs without requiring additional reagents at the point of use [20–22].

Herein, interlayer-confined redox (ICR) MXene laminates are presented as a materials-centric solution to these challenges. In this design, Ti_3_C_2_T_*x*_ sheets are co-filtered with MB, allowing the mediator to be immobilized within the MXene galleries via π–π and electrostatic interactions, forming a delocalized solid-state redox network that produces a sharp, built-in voltammetric signal. This approach confines the mediator within the layered structure, suppressing mediator diffusion while maintaining electrochemical accessibility. The freestanding laminate allows direct transfer onto screen-printed electrodes, enabling reagent-free detection without soluble mediators or post-assembly surface functionalization. The designed platform’s sensing capability is evaluated against erdafitinib (ERD), an oral FGFR inhibitor approved for the treatment of metastatic urothelial carcinoma [23]. ERD is a clinically important target for therapeutic drug monitoring (TDM); however, current quantification relies on HPLC-UV or LC-MS/MS, which, despite excellent analytical performance, require specialized laboratories, organic solvents, and trained personnel, thereby limiting decentralized testing. Reported plasma concentrations of ERD generally range from 0.1 to 2 µM, while urinary concentrations can reach several micromolar levels, depending on dose and pharmacokinetics [24, 25]. Therefore, analytical coverage from low nanomolar to micromolar concentrations is essential for practical therapeutic drug monitoring and biofluid analysis. In addition to its clinical significance, ERD belongs to the broader class of targeted anticancer agents that are increasingly discussed as pharmaceuticals of emerging concern in hospital effluents and aquatic environments [26]. A portable, reagent-free electrochemical assay integrated with SPEs would therefore complement established methods by enabling rapid checks in point-of-care or near-patient settings and, in principle, could be adapted for on-site surveillance of anticancer drugs in complex water matrices.

The work demonstrates that ICR laminates deliver a stable intrinsic MB peak ( ≈ − 0.25 V) that is quantitatively suppressed by ERD across the 0.05–10.5 µM range with a 0.01 µM LOD, while maintaining ≤ 0.8% RSD for intra-/inter-electrode precision and 98–102% recoveries in spiked human urine using a straightforward SPE format. The mechanism involves mediator-gated inhibition, in which ERD interaction near accessible gallery/interfacial regions blocks or perturbs the interlayer-confined MB redox pathway, as supported by electrochemical impedance spectroscopy (EIS) and density functional theory (DFT) analyses. This solid-state design eliminates the need for soluble mediators or complex immobilization, embedding the mediator within Ti_3_C_2_T_*x*_ laminates. This materials pathway also supports the wider relevance of MXene-based sensors for field analysis in complex aqueous environments, including resource-processing and tailings-impacted waters, where portable and low-reagent electrochemical platforms are increasingly attractive. 

## Materials and methods

### Reagents and materials

Titanium aluminum carbide (Ti_3_AlC_2_, MAX phase), lithium fluoride (LiF, analytical grade), hydrochloric acid (HCl, 37% w/w), methylene blue (MB), phosphate-buffered saline (PBS), and ethanol were purchased from Sigma-Aldrich (Germany) and used as received. Deionized (DI) water (18.2 MΩ cm) was used in all preparations. Commercial screen-printed electrodes (SPEs) (Zensor, Taiwan) with a 5 mm carbon working electrode, carbon counter electrode, and an on-chip Ag pseudo-reference electrode were used for all electrochemical measurements. Nafion (5 wt% in lower aliphatic alcohols, Sigma-Aldrich) was diluted to 0.5% (w/v) in isopropanol and employed as a thin topcoat for mechanical retention of the laminate during immersion.

### Preparation of interlayer-confined redox (ICR) MXene laminates

To embed a delocalized, solid-state mediator network within the galleries, the as-prepared Ti_3_C_2_T_*x*_ dispersion (5 mg mL^− 1^) was mixed with an MB working solution (18 µM) under N_2_ for 45 min to allow adsorption/intercalation. The resulting suspension was vacuum-filtered through a Celgard membrane (low pressure) to yield free-standing laminates, which were dried at room temperature. Batch labels ICR-MB-MX-*x* denote the retained MB loading in the film (*x* ≈ 4, 7, 11, 14 µM), quantified by UV − Vis spectrophotometry of the filtrate at λ = 650 nm against an MB calibration curve (Fig. S1).

### Assembly of reagent-free laminate sensors on SPEs

Circular disks (5 mm) were punched from the ICR-MB-MX-*x* laminates and transferred directly onto the carbon working area of the SPEs. The disks were fixed in place by pressing at ~ 50 psi in a single-stroke press to ensure intimate contact. To provide additional mechanical robustness during immersion, a 0.5% (w/v) Nafion solution was drop-cast sparingly onto the laminate surface and allowed to dry at room temperature.

### Characterizations

UV–Vis studies were performed with a UV-3600 ultraviolet–visible spectrophotometer (Shimadzu). The surface morphology was examined using a scanning electron microscope (MAIA3 XMU, Tescan) and a field-emission transmission electron microscope (FEI Tecnai G2 F30). X-ray diffraction (XRD) patterns were collected on an Ultima IV diffractometer (Rigaku). X-ray photoelectron spectroscopy (XPS, ESCALAB 250, Thermo VG, USA) was used to investigate changes in surface chemical states. Three-dimensional excitation–emission matrix (3D-EEM) (FL 8500 Fluorescence Spectrophotometer) was recorded to confirm the distribution and optical signatures of MB within the laminates. Static water contact angles were measured on ICR-MB-MX films using a Krüss Drop Shape Analyzer (DSA). ζ-Potential measurements of Ti_3_C_2_T_*x*_ and MB-loaded dispersions were carried out using a nanoparticle-size zeta potential analyzer (NaNoZS, UK). The pH measurement is performed with a pH meter (PH-B200E Infitek Co., Ltd.).

### Electrochemical characterizations

All electrochemical measurements were performed on a CHI600F Series potentiostat (CH Instruments, Inc.) with ICR-MB-MX-*x*/SPE electrodes, which included a working, a counter, and an on-chip Ag pseudo-reference electrode. The electrode was easily set up for measurement using a custom SPE holder connected to a potentiostat using alligator clips. Experiments were carried out in PBS (0.01 M) (pH 6.0) at room temperature. Cyclic voltammetry (CV) was recorded in the potential range − 0.5 to 0.1 V with a current sensitivity of 1.0 × 10^− 5^. Differential pulse voltammetry (DPV) was performed over − 0.4 to 0.1 V with a pulse amplitude of 0.075 V and a pulse width of 0.065 s. EIS was recorded at the formal potential of the MB couple with a 10 mV AC perturbation over a frequency range of 0.1 Hz to 100 kHz. Calibration and analytical measurements were performed using a custom SPE connector, with a new ICR‑MB‑MX‑11/SPE used for each ERD concentration (*n* = 3 per point). ICR‑MB‑MX‑11 disks were cut to 5 mm diameter and selected to have nominally identical areal loading (same mass per disk), ensuring that variations in ΔI and R_ct_ arose from analyte concentration rather than electrode reuse or differences in MXene/MB mass.

For analytical sensing, ERD solutions of various concentrations were freshly prepared in PBS (0.01 M, pH 6.0). A 10 µL drop was placed on the ICR-MB-MX-11 electrode and incubated for 5 min at room temperature to allow interaction with the MB-MXene laminate. Subsequently, DPV was recorded directly in the same droplet without an external redox mediator. The blank MB signal was first measured in PBS, and the response was calculated based on the peak currents before and after ERD, as ΔI = I_0_ − I_ERD_, where I_0_ and I_ERD_ are the MB peak currents before and after ERD exposure, respectively. Urine samples were diluted 1:1 with PBS (pH 6.0) and spiked with ERD. A 10 µL sample was applied to ICR-MB-MX-11, incubated for 5 min, and then measured via DPV.

## Results and Discussion

### Structural and surface characterization of ICR-MB@MXene laminates

A fresh Ti_3_C_2_T_*x*_ dispersion was prepared from Ti_3_AlC_2_ MAX phase via selective etching, and the structural transformation was first confirmed by TEM. The pristine Ti_3_AlC_2_ MAX phase exhibits a dense, layered morphology with compact stacking, as shown in Fig. S2a, which serves as the precursor for MXene formation. After etching and delamination, TEM reveals thin, transparent Ti_3_C_2_T_*x*_ flakes with well-defined lateral dimensions (Fig. S2b), confirming successful exfoliation into few-layer MXene sheets suitable for laminate assembly. To embed a delocalized redox network, the Ti_3_C_2_T_*x*_ dispersion was mixed with MB under N_2_ for 45 min, followed by vacuum filtration to yield free-standing laminates, which were then used to prepare ICR-MB-MX-*x* films. The morphology of the assembled films was examined after transfer onto SPEs. Compared with drop-cast MXene film, which exhibits discontinuous coverage with visible pinholes and surface ruptures (Figure S3(a)), indicating poor film integrity and non-uniform electrical contact, the representative ICR-MB-MX-11 laminate forms a dense, continuous network with significantly fewer surface defects (Fig. S3b), demonstrating improved structural cohesion and interfacial contact due to the vacuum-filtered laminate architecture.

**Fig. 1:**
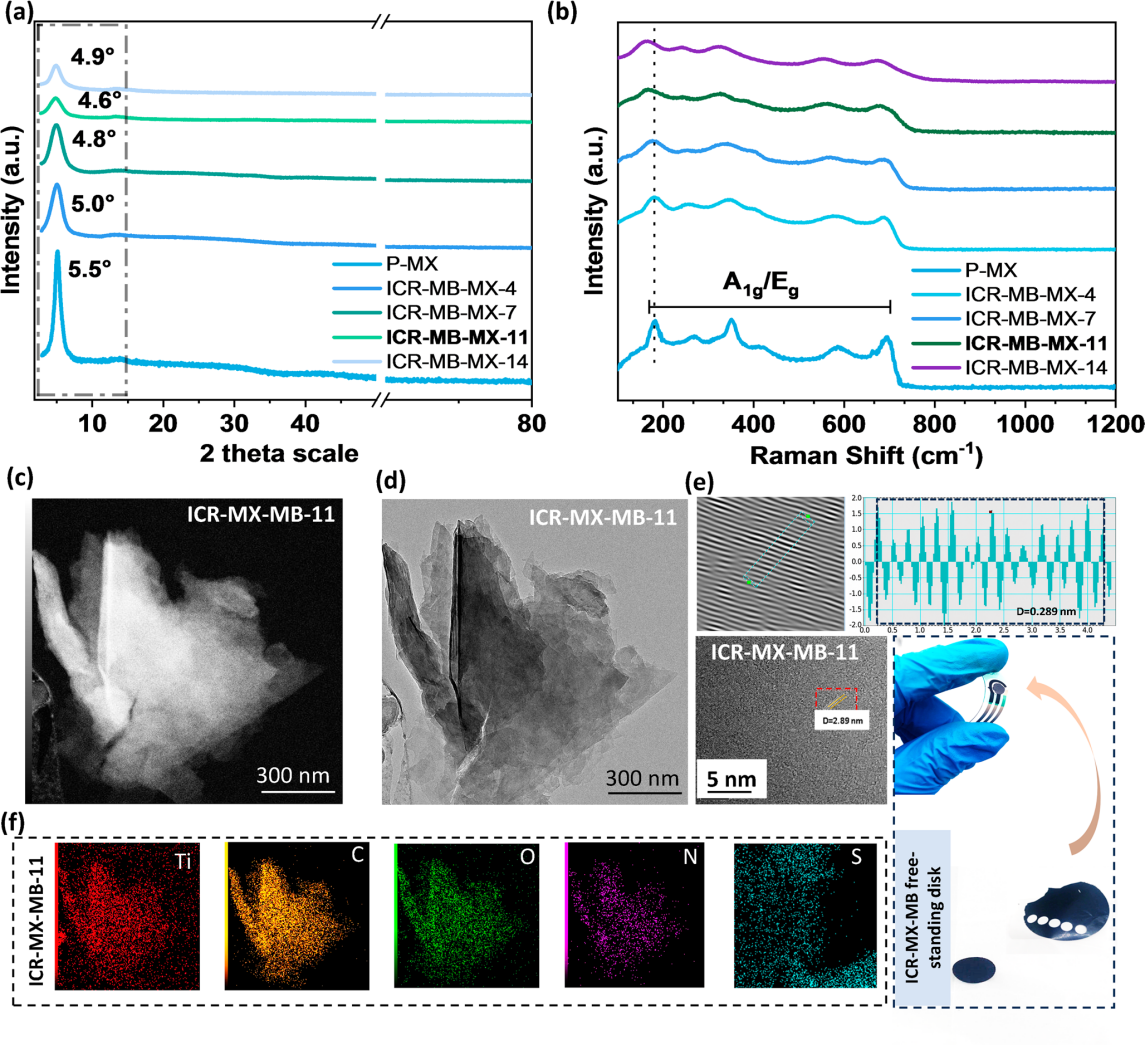
(**a**) XRD patterns, and (**b**) Raman profiles of ICR-MB-MX-11 in reference to pristine MXene and other compositional counterparts, (**c**-**d**) TEM and dark-field TEM images of ICR-MB-MX-11 illustrating layered flake morphology, (**e**) High-resolution TEM with corresponding FFT/line-profile showing an in-plane lattice periodicity (**f**), STEM-EDS elemental maps (Ti, C, O, N, S) confirming uniform through-thickness distribution of MB, rather than surface adsorption, with the right panel showing a photograph of the free-standing ICR-MB-MX-11 laminate disk over SPE assembly, highlighting mechanical integrity and transferability

XRD analysis confirms that vacuum-filtered Ti_3_C_2_T_*x*_ laminates retain the MXene framework after MB intercalation, with a measurable expansion of the interlayer galleries. The (002) reflection shifts from 2θ ≈ 5.5° (pristine) to 4.6–4.9° for ICR-MB-MX-*x* series while higher‑order (00 L) peaks remain, evidencing intercalation without loss of lamellar order. Using Cu Kα (λ = 1.5406 Å), the corresponding basal spacings are d_002_ ≈ 1.61 nm pristine Ti_3_C_2_T_*x*_ (P‑MX), 1.92 nm (ICR-MB‑MX‑11), and 1.80 nm (ICR-MB‑MX‑14) [27]. Notably, no anatase/rutile reflections are observed, indicating that mediator loading does not crystallize TiO_2_ during film formation (Fig. 1a) [28]. Raman spectra further confirm the preservation of the MXene’s A_1g_/E_g_ modes (≈ 196, 293, 391, 712 cm⁻¹) after MB loading, with small red‑shifts (≈ 2 cm^− 1^) and modest attenuation that grows with MB content. The conserved phonon pattern and lack of new oxide bands in 200–1000 cm^− 1^ confirm that interlayer MB couples electronically to the lattice without introducing any crystalline oxide phases (Fig. 1b) [29–31]. Figure 1c, d shows dark-field TEM and TEM images with an in-plane view of representative ICR-MB-MX-11 that shows maintained MXene morphology along with well-ordered lattice fringes. The 2D-FFT and line-intensity profile from the boxed region (Fig. 1(e), top-right panel) resulted in a fundamental spatial frequency of ≈ 3.46 nm^− 1^, corresponding to a real-space periodicity of 0.289 ± 0.01 nm (d = 1/q). The 0.289 nm spacing matches the Ti − C sublattice periodicity reported for Ti_3_C_2_T_*x*_, confirming the preservation of in-plane crystallinity [32, 33]. The coexistence of a constant in‑plane periodicity (~ 0.289 nm) with an expanded d_002_ (~ 1.61–1.92 nm) indicates that MB expands the galleries while preserving in‑plane crystallinity. STEM‑EDS shows N distributed across the flake area, consistent with MB incorporation throughout the laminate rather than isolated surface aggregates (Fig. 1f). The bottom-right panel shows the free-standing ICR-MB-11 laminate disk over the SPE assembly, highlighting its ease of transferability.

**Fig. 2:**
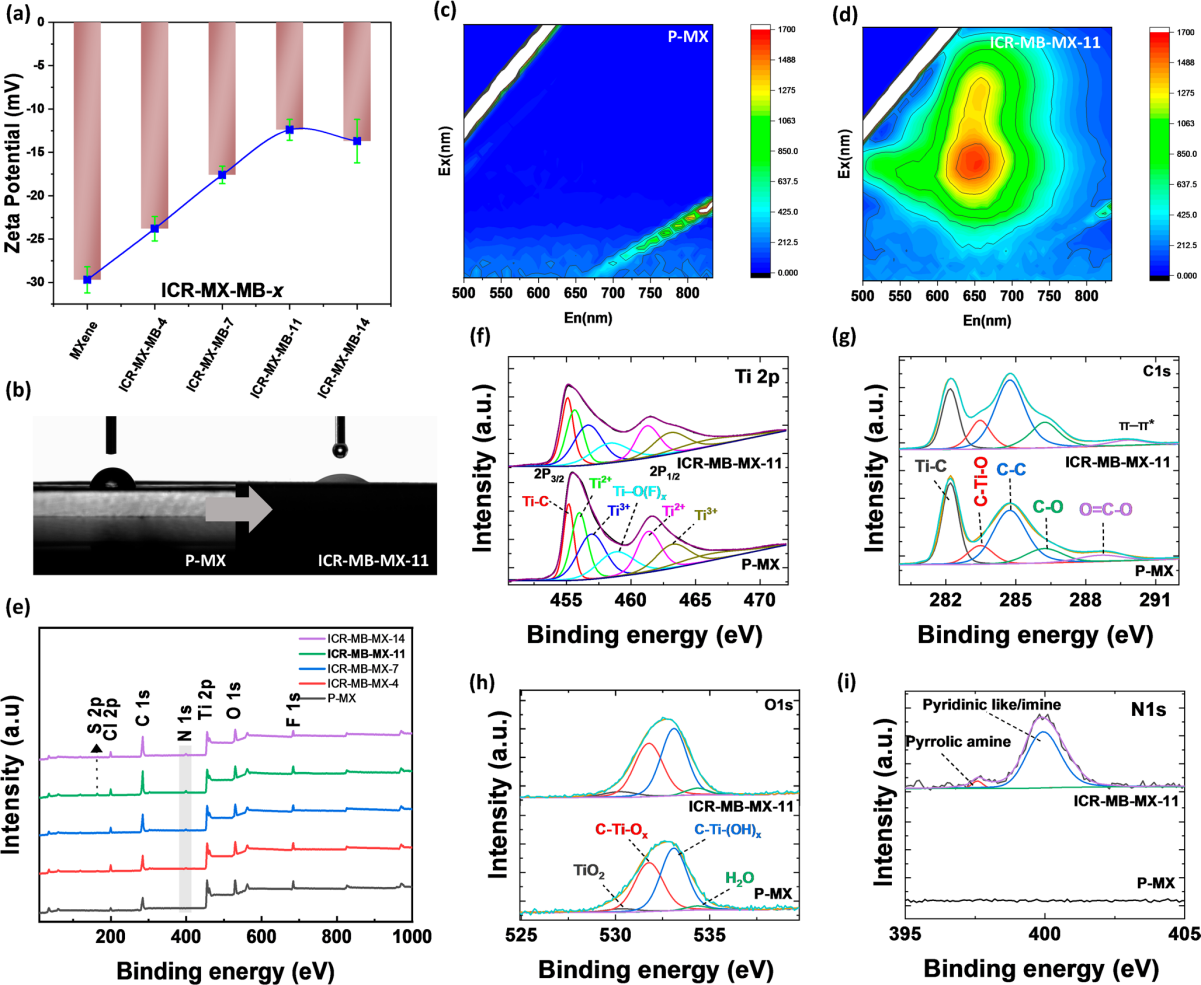
(**a**) ζ‑Potential of pristine Ti_3_C_2_T_*x*_ (P‑MX) and ICR-MB‑MX-*x* dispersions (**b**, **c**) 3D (EEM) maps of P‑MX and ICR‑MB‑MX‑11 laminates, (**d**) Static water contact‑angle images for P‑MX and ICR‑MB‑MX‑11, indicating increased surface hydrophilicity after MB intercalation, (**e**) XPS Survey spectrum of ICR-MB-MX-*x*, (**f**, **g**) High‑resolution Ti 2p and C 1s XPS spectra comparison of P‑MX and ICR‑MB‑MX‑11, confirming preservation of the Ti–C framework and the appearance of aromatic π‑features from MB, and corresponding (**h**, **i**) O 1s and N 1s spectra for ICR‑MB‑MX‑11

The zeta-potential trend reflects dispersion-phase charge screening within ICR-MB-MX-*x* (Fig. 2a). In PBS, the ζ-potential shifts from − 29.7 ± 0.6 mV (pristine) to − 23.8 ± 0.7 mV (ICR-MB-MX-3), − 17.6 ± 0.6 mV (ICR-MB-MX-8), and − 12.4 ± 0.5 mV (ICR-MB-MX-11) (*n* = 3), evidencing electrostatic binding without charge reversal [34]. The trend is consistent with the 002-downshift observed by XRD, which, together, supports interlayer confinement as the dominant binding motif [35]. Since MB serves as the built-in redox probe, EEM is used to confirm its optical signature in the solid laminate [36]. The pristine Ti_3_C_2_T*x* film shows negligible fluorescence across the scanned window (baseline), consistent with a metallic, low-gap conductor (Fig. 2b). In contrast, ICR-MB-MX-11 exhibits a single, intense ridge centered at E_*x*_ ≈ 650 nm and E_*m*_ ≈ 650–700 nm (Fig. 2c, d), matching the canonical fingerprint of monomeric MB in dilute media [37]. The absence of secondary shoulders suggests that MB exists predominantly as monomeric or non-bonding associated species under these confined conditions [38]. Static water contact‑angle measurements show that θ decreases from ≈ 45.0 ± 1.5° for P‑MX to ≈ 30.0° ± 1.2° for the ICR‑MB‑MX‑11 laminate, indicating that interlayer MB confinement slightly increases surface hydrophilicity and is consistent with the ζ‑potential neutralization and EEM evidence for uniform dye incorporation [39].

The chemical state of the ICR-MB-MX-*x* is resolved by XPS. Wide-scan XPS confirms that MB loading modifies only the near-surface composition expected for interlayer-confined redox (ICR) laminates, without introducing crystalline TiO_2_ [40]. Compared with pristine Ti_3_C_2_T_*x*_, ICR-MB-MX-*x* films retain the Ti 2p, C 1s, O 1s, and F 1s envelopes but gain a clear N 1s signal whose intensity increases with *x* (MB-MX-4 < 7 < 11 < 14), and a weak Cl 2p doublet attributable to the chloride counter-ion of methylene blue (Fig. 2(e)). The C/O envelope becomes richer in carbon with loading (π-rich MB backbone), while F 1s remains essentially unchanged. No new features appear in the 458.8 eV region that would indicate crystalline TiO_2_, aligning with the XRD wide-angle patterns. High-resolution Ti 2p (Fig. 2f) was fit using a Shirley background, a 2:1 2p_3/2_:2p_1/2_ area ratio, and 5.7–5.8 eV spin–orbit splitting. Four components are resolved: Ti–C (≈ 454.6 eV), Ti^2+^ (≈ 455.4 eV), Ti^3+^ (≈ 456.5 eV), and Ti–O(F)_*x*_ (≈ 458.0 eV), attributed to termination-bonded Ti at the outermost layers of MXene (Figure S2). Upon MB confinement, all Ti components shift ≈ 0.2 eV to lower binding energy, consistent with strengthened Ti–π interactions and modest electron density redistribution [41]. Crucially, the 458.8 eV feature, typical of anatase/rutile TiO_2_, is absent, corroborating that no crystalline oxide forms during laminate fabrication [42]. C 1s spectra (Fig. 2g) were referenced to C–C/C = C at 284.8 eV. Pristine films show dominant Ti–C (≈ 282.2 eV) with minor C–Ti–O (≈ 283.5) and overlayer carbon (C–C/C = C, ≈ 284.8 eV, O = C–O ≈ 288.8 eV). In ICR-MB-MX-11, the sp^2^ C–C/C = C envelope resembles that of Ti–C, and a distinct π–π satellite appears at ≈ approximately 289.9 eV, a characteristic fingerprint of aromatic systems [43]. The persistence of a strong Ti–C component confirms that the carbide framework remains intact, while the π features report the embedded dye network [44]. O 1s deconvolution (Fig. 2h) shows C–Ti–O (≈ 531.8), C–Ti–(OH)_*x*_ (≈ 533.1 eV), and physiosorbed H_2_O (≈ 534.4 eV). With MB loading, the hydroxyl fraction rises modestly, consistent with: (i) electrostatic/π–π association of cationic MB within − O/−OH-terminated galleries and (ii) slightly higher surface hydration around the confined dye [16]. A sharp TiO_2_ 530.4 eV feature is not observed, agreeing with the Ti 2p and XRD conclusions that crystalline oxide is absent [41]. Pristine Ti_3_C_2_T_*x*_ shows no N 1s signal (Fig. 2i), whereas ICR-MB-MX-11 exhibits a prominent N 1s peak, which can be fit with contributions that can be decomposed into two components at 399.5 and 402.1 eV, consistent with different nitrogen environments within the MB framework [41].

### Electrochemical evaluation of the ICR-MB-MX-*x* electrodes

**Fig. 3:**
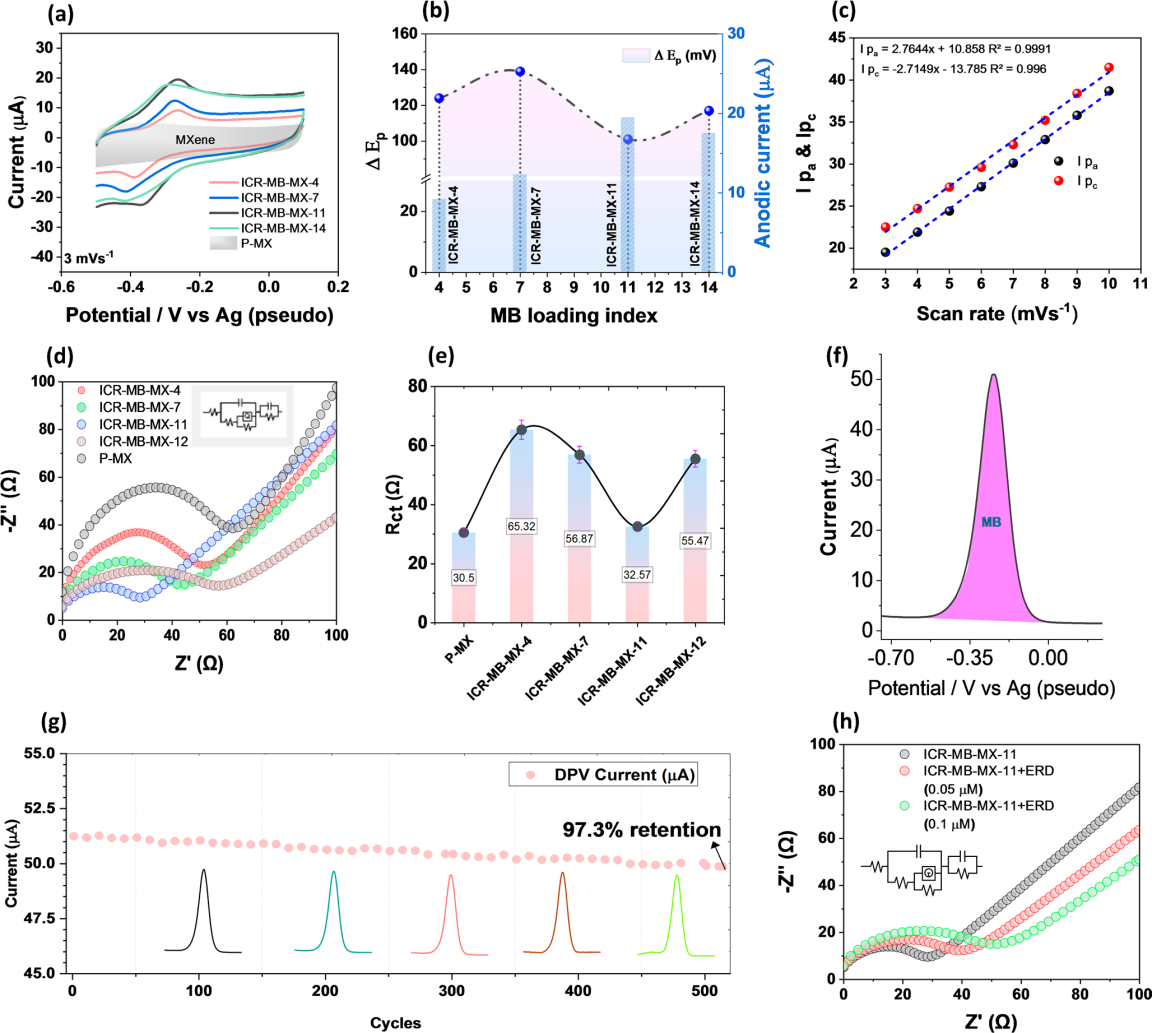
Electrochemical characterization of ICR-MB-MX-*x* electrodes in PBS (pH 6.0), (**a**) low-scan-rate CVs of pristine Ti_3_C_2_T_*x*_ and ICR-MB-MX-*x* electrodes, (**b**) ΔE_p_ comparison for different MB loadings, (**c**) scan-rate-dependent kinetic analysis of ICR-MB-MX-11 (**d**) Nyquist plots with fitted equivalent circuit, (**e**) extracted R_ct_ values, (**f**) DPV response of the confined MB probe, (**g**) DPV cycling stability, and (**h**) ERD-induced EIS response

CV measurements were performed at low scan rates to minimize polarization effects and clarify the charge-storage kinetics of the ICR-MB-MX-*x*. In PBS (pH 6.0), pristine Ti_3_C_2_T_*x*_ exhibits a predominantly capacitive response without a distinct redox couple, whereas the ICR-MB-MX-*x* laminates display a well-defined MB-related redox couple centered around − 0.25 V vs. the on-chip Ag pseudo-reference, consistent with the MB/MB⁺ redox level reported in previous studies (Fig. 3a) [45, 46]. This confirms that MB introduces an electrochemically addressable interlayer redox center. Compared to pristine Ti_3_C_2_T_*x*_, MB-intercalated electrodes exhibit more polarization due to partial blockage of transport pathways, with the enhanced signal mainly from MB redox activity rather than improved conductivity. The influence of MB loading was further evaluated from peak separation and peak current at a low scan rate. The ΔE_p_ values for ICR-MB-MX-4, ICR-MB-MX-7, ICR-MB-MX-11, and ICR-MB-MX-14 are 124, 139, 101, and 117 mV, respectively (Fig. 3b). Among these, MB-MX-11 exhibits the smallest ΔE_p_ with improved redox response, indicating an optimal balance between redox-site density and charge-transfer accessibility. In contrast, higher loading (ICR-MB-MX-14) results in relatively broader peaks and slightly reduced current, suggesting interlayer crowding and increased transport limitation [46]. The charge-storage kinetics were analyzed using scan-rate-dependent CVs of ICR-MB-MX-11 in the range of 3–10 mV s^− 1^ (Fig. S4a and 3c). The anodic and cathodic peak currents increase linearly with scan rate, following Ip_a_ = 2.764*v* + 10.858 (R^2^ = 0.9991) and Ip_c_ = 2.714*v* + 13.785 (R^2^ = 0.9960), respectively. A relatively weaker linear dependence is observed for I_p_ vs. *v*^1/2^, while log I_p_-log *v* fitting yields b-values of 0.577 and 0.510 for the anodic and cathodic peaks, respectively (Fig.  S4c, d). These results indicate mixed interlayer-confined redox kinetics with a significant transport-controlled contribution, together with quasi-reversible charge transfer and scan-rate-dependent polarization within the MB-confined MXene galleries [47].

To further benchmark the electrochemically accessible area and outer-sphere electron-transfer kinetics, Ru(NH_3_)_6_^3+/2+^ (5 mM) was used as a redox probe because its redox process occurs at negative potentials, avoiding the risk of positive-potential oxidation on Ti_3_C_2_T_*x*_-based electrodes [48]. CVs were recorded for pristine Ti_3_C_2_T_*x*_/SPE and ICR-MB-MX-11 at different scan rates from 5 to 100 mV s^− 1^ (Figure S5). For both electrodes, the anodic and cathodic peak currents increased linearly with *v*^1/2^, confirming diffusion-controlled outer-sphere redox behavior. The apparent electrochemically active surface area (A_ECSA_) was calculated from the Randles–Ševčík equation:

$$\;I_{p}=\;\mathrm{2.69}\times\;10^{5}n^{3/2}AD^{1/2}Cv^{1/2}$$

Where I_p_ is the peak current, *n* = 1, D = 8.43 × 10^− 6^ cm^2 ^s^− 1^, C = 5.0 × 10^− 6^ mol cm^− 3^, and *v* (Vs^− 1^) is the scan rate. Accordingly, pristine P-MX exhibits an apparent A_ECSA_ of 0.118 cm^2^, while ICR-MB-MX-11 showed a lower value of 0.091 cm^2^. This decrease indicates that MB confinement partially occupies accessible interlayer sites and limits the access of the external Ru(NH_3_)_6_^3+^/^2+^ probe, while the retained response confirms that the laminate remains electrochemically accessible. The apparent heterogeneous electron-transfer rate constant ($k^{0}_{app}$) was estimated using the Nicholson method:

$$k^{0}_{app}=\psi\;\mathrm{(}\frac{\pi\;DnFv}{RT}{)}^{1/2}$$

Where k^0^_app_ is the apparent heterogeneous electron-transfer rate constant, ψ is the Nicholson kinetic parameter obtained from the peak separation(ΔE_p_), D is the diffusion coefficient of Ru(NH_3_)_6_^3+^/^2+^, n is the number of transferred electrons, F is the Faraday constant, *v* is the scan rate, R is the gas constant, and T is the absolute temperature. The average k^0^_app_ values were 3.45 × 10^− 3^cm s^− 1^ for P-MX and 4.78 × 10^− 4^cm s^− 1^ for ICR-MB-MX-11. The lower A_ECSA_ and k^0^_app_ after MB confinement confirm partial blocking of external probe-accessible pathways. Importantly, this behavior is fully consistent with the sensing principle of ICR-MB-MX-11, in which the analytical DPV response is governed by the internal, interlayer-confined MB redox network rather than by enhanced accessibility to an external redox probe.

EIS measurements were further performed to evaluate how MB loading affects interfacial charge-transfer resistance and transport accessibility within the laminate (Fig. 3d). As shown, pristine Ti_3_C_2_T_*x*_ exhibits the lowest R_ct_ due to its highly conductive, mediator-free MXene network. After MB intercalation, R_ct_ increases relative to pristine Ti_3_C_2_T_*x*_, confirming that MB partially obstructs electronic/ionic transport pathways within the laminate. However, among the MB-loaded electrodes, R_ct_ shows a loading-dependent trend: ICR-MB-MX-4 and ICR-MB-MX-7 exhibit increasing resistance, whereas ICR-MB-MX-11 exhibits the lowest R_ct_ among the ICR series (Fig. 3e). This indicates that moderate MB loading improves redox connectivity and charge-transfer accessibility without excessive interlayer blockage [49]. At higher loading, ICR-MB-MX-14 shows increased R_ct_, consistent with overfilling of the galleries and partial transport limitation. Together with EEM and ζ-potential neutralization, the EIS baseline identifies ICR-MB-MX-11 as the optimal composition for sensing, maximizing electron transfer efficiency without hindering transport.

To translate the high baseline current into analytical sensitivity, differential-pulse voltammetry (DPV) was used to generate the primary signal [50]. DPV recorded on ICR-MB-MX-11 exhibits a single sharp peak at ≈ − 0.25 V (Fig. 3f). As detailed in the SI optimization (pulse amplitude ≈ 0.075 V; width ≈ 0.065 s), DPV suppresses capacitive current and amplifies the mediator signal, enabling sensitive detection of ERD without adding soluble reagents (Figure S6) [51].

To verify that the mediator remains interlayer-confined, 500 consecutive DPV scans were performed on the same ICR-MB-MX-11 SPE in PBS (pH 6.0), and the MB peak current was monitored as a function of cycle number (Fig. 3g). The MB peak retains 97.3% of its initial current after 500 DPV cycles, and UV–Vis analysis (Figure S7) of the electrolyte reveals MB levels near the detection limit, indicating negligible leaching under operating conditions. When ERD is introduced, the Nyquist semicircle at $\:{\mathrm{E}}^{{\mathrm{0}}^{{\prime\:}}}$expands systematically, indicating a progressive slowdown of interfacial kinetics (Fig. 3h). The charge-transfer resistance increases from 35.8 Ω for ICR-MB-MX-11 to 50.2 Ω in the presence of 0.05 µM ERD and 65.5 Ω at 0.10 µM ERD. This dose-dependent rise in $\:{\mathrm{R}}_{\mathrm{ct}}$ is fully consistent with the suppression of the MB DPV peak and supports a mediator-gated inhibition mechanism, in which ERD interaction within the galleries perturbs the interlayer redox network, generating a concentration-dependent inhibition signal proportional to the ERD concentration [46].

### Analytical detection of erdafitinib (ERD)

**Fig. 4:**
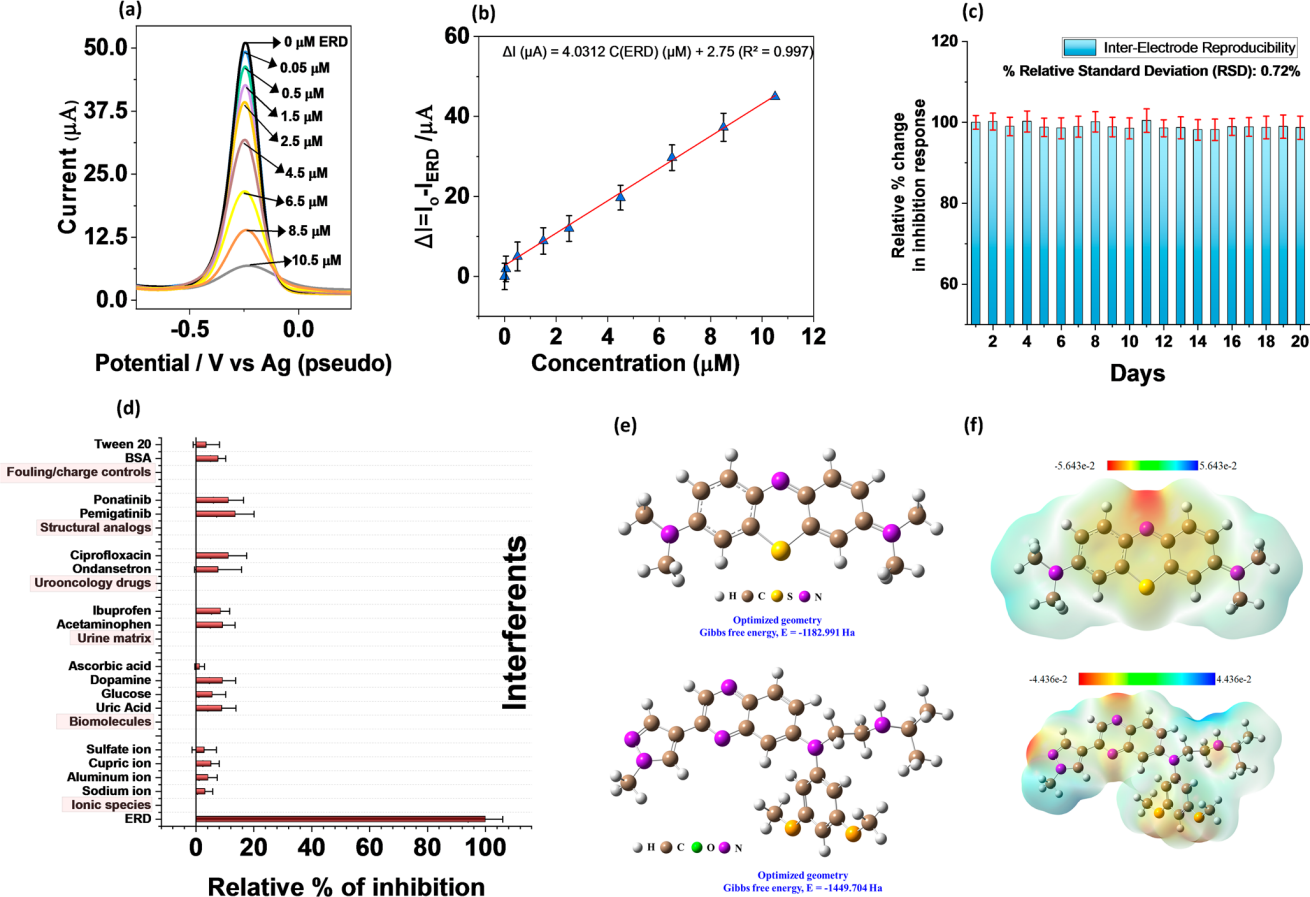
(**a**) Representative DPV based inhibition response of ICR-MB-MX-11 towards different concentrations of ERD, and (**b**) the calibration curve representing an increase in current (ΔI = I_o_−I_ERD_ ) with rising ERD concentration, (**c**) inter-electrode reproducibility of the ICR-MB-MX-11 electrode during 20 days of storage, (**d**) relative change in inhibition response with added interferents, and (**e**-**f**) electron-density mapping highlighting the electron distribution within MB, and ERD molecule with red for high electron density and blue for low electron density

Under the optimized DPV conditions, the ICR-MB‑MX‑11 laminate displays a single, well‑defined anodic peak at ≈ − 0.25 V vs. on‑chip Ag (Fig. 4a). For the calibration in Fig. 4(b), each ERD concentration was measured on a freshly prepared ICR-MB-MX-11. For each electrode, the baseline MB signal was first recorded in blank PBS, followed by exposure to ERD at a single target concentration [52]. The analytical signal is defined as ΔI (ΔI defined as the absolute current change) = I₀ – I_ERD_, where I₀ and I_ERD_ are the MB peak currents before and after ERD addition, respectively (*n* = 3). Upon successive additions of ERD (0.05–10.5 µM), the peak current decreases, consistent with inhibition of a surface‑confined mediator rather than formation of a new redox couple [41]. Over 0.05–10.5 µM, ΔI increases linearly with ERD concentration (Fig. 4b), following: ΔI (µA) = 4.0312 C(ERD) (µM) + 2.75 (R^2^ = 0.997), affording a 0.01 µM limit of detection (3σ/slope). The working pH window was examined from 3 to 9 (Figure S8). The inhibition response is maximal and most reproducible at pH 6.0. The pH dependence likely reflects a combination of ERD speciation and MXene surface charge, where near pH 6, ERD remains sufficiently protonated to interact with the negatively terminated galleries while maintaining good solubility. At more acidic or basic pH levels, changes in ERD charge and surface termination states reduce the effective interaction strength, resulting in smaller inhibition signals [36, 53, 54]. The electrode displayed excellent stability and signal reproducibility with independently fabricated disks stored for 20 days, achieving an inter-electrode RSD of 0.72% and, over 10 consecutive measurements, an RSD of 0.77% (Fig. 4c, S9).

To clarify the intrinsic selectivity of the ICR-MB-MX-11, selectivity measurements were performed using Nafion-free ICR-MB-MX-11 electrodes. Representative interferents were grouped into (i) ionic species (Na^+^, Al^3+^, Cu^2+^, SO₄^2−^), (ii) biomolecules and electroactive metabolites (glucose, dopamine, ascorbic acid, uric acid), (iii) urine-matrix components (creatinine, phosphate), (iv) uro-oncology co-medications (acetaminophen, ibuprofen, ondansetron, ciprofloxacin), (v) structural analogues (pemigatinib, ponatinib), and (vi) fouling/charge-control agents (BSA, Tween-20) (Fig. 4d). Each interferent was tested at a concentration 10 times that of ERD. Under Nafion-free conditions, the interferents produced only minor changes in the MB inhibition response with ΔI remaining within approximately 15% (*n* = 3) of the ERD-normalized signal, whereas ERD produced the dominant response defined as 100%. The DPV curves for ascorbic acid are presented in Fig. S10 as a representative interferent to demonstrate the reliability of the DPV signal, particularly with respect to peak shape and width. The minimal change in peak characteristics confirms the platform’s analytical robustness. Ascorbic acid does not significantly affect the MB peak at around − 0.25 V, nor does it produce overlapping signals, indicating the ERD response is not due to nonspecific oxidation. Thus, the selectivity mainly stems from the ICR-MB-MX architecture rather than Nafion permselectivity. The response is caused by three combined factors: (i) the MB redox signal is detected at a potential that is largely unaffected by the tested interferents; (ii) only molecules with suitable affinity and molecular dimensions can effectively access or interact with the negatively charged, π-rich MXene galleries; and (iii) ERD uniquely perturbs the interlayer DOS (discussed later), as evidenced by the rise in R_ct_ in EIS (Fig. 3h), while other species either do not enter the galleries or couple relatively weakly.

Finally, ERD spiked into pre-treated human urine (1:1 dilution in PBS) was quantitatively recovered, yielding recoveries of 98.0–102.0% with an RSD ≤ 3.3% (*n* = 3) across the tested concentration range (Table S1). The representative DPV curves for the spiked samples show negligible variation in peak shape and intensity, indicating stable signal response and minimal matrix-induced distortion (Fig. S11). Combined with the low LOD, good inter-electrode reproducibility, and limited signal variation in the presence of complex biological components, these results demonstrate the matrix tolerance and analytical feasibility of the ICR-MB-MX-11 laminate as a reagent-free solid-state platform for ERD detection in urine samples [54].

Furthermore, comparison with previously reported ERD detection methods (Table S2) confirms that the present platform provides a competitive linear detection range and detection limit, while eliminating the need for external redox mediators or complex surface functionalization strategies. From a practical cost perspective, sensor cost is mainly determined by the commercial SPE substrate, which typically costs USD 3–5 per disposable carbon electrode, depending on the supplier and purchase quantity. The ICR-MB-MX layer adds only a minor material cost because it uses a small laminate disk and a low amount of MB, while eliminating the need for expensive biological recognition elements, such as noble-metal labels, enzymes, antibodies, or aptamers, resulting in a simpler, more material-efficient fabrication process. Simultaneously, reagent consumption during operation is reduced because the redox mediator is pre-confined within the MXene laminate, removing the necessity for soluble mediators during measurement. Detection occurs in aqueous PBS with a microliter-scale sample volume, avoiding the use of organic mobile phases and the extensive sample preparation typically associated with chromatographic ERD assays. Although MXene synthesis involves wet-chemical etching, the final sensing procedure is reagent-free, low-volume, and suitable for portable electrochemical analysis.

### Mechanistic interpretation and DFT calculation

**Fig. 5:**
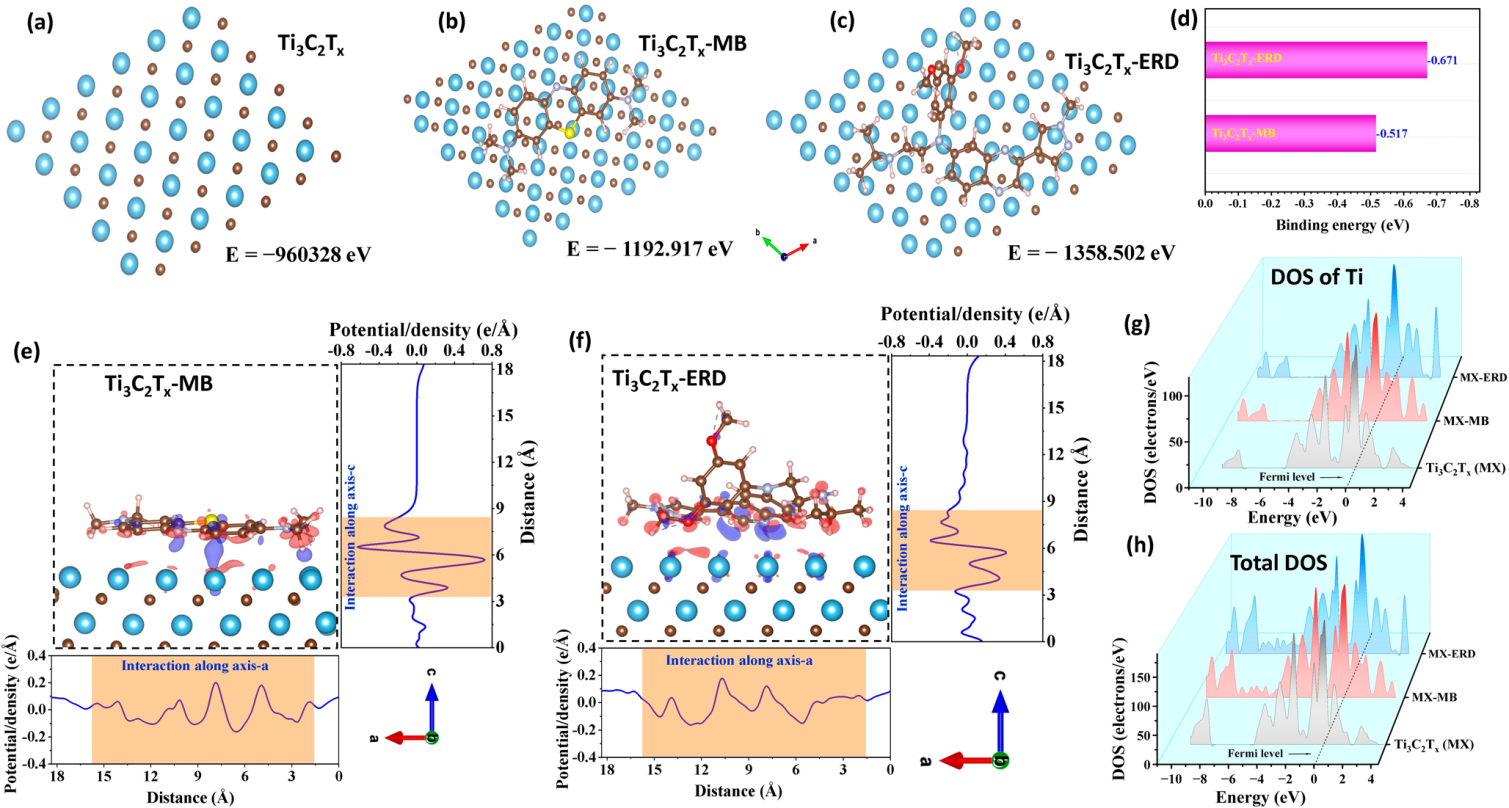
(a-c) optimized total energy geometry visualization of Ti_3_C_2_T_*x*_, Ti_3_C_2_T_*x*_-MB, and Ti_3_C_2_T_*x*_*-*ERD system (d) Binding energy for Ti_3_C_2_T_*x*_-ERD and Ti_3_C_2_T_*x*_-MB system, (e-f) models depicting the charge energy distribution for Ti_3_C_2_T_*x*_-MB and Ti_3_C_2_T_*x*_-ERD system, (g-h) DOS of Ti-atoms and total density of states (TDOS) of Ti_3_C_2_T_*x*_ surface, Ti_3_C_2_T_*x*_-MB, and Ti_3_C_2_T_*x*_-ERD system

The combined electrochemical and DFT results substantiate a mediator-gated modulation of the density of states (DOS) mechanism for ERD detection within the MB-intercalated Ti_3_C_2_T_*x*_ laminate. Geometry optimizations place MB essentially flat on the − O/−F/−OH‑terminated Ti_3_C_2_T_*x*_ basal plane with a calculated binding energy of − 0.517 eV and an equilibrium separation of ≈ 2.2 − 2.4 Å, indicative of strong interfacial binding that preserves the underlying carbide lattice (Fig. 5a-d) [55]. Charge‑density‑difference isosurface reveals broad, delocalized regions of charge accumulation and depletion at the MB–MXene interface (Fig. 5e), and the Ti‑projected DOS for Ti_3_C_2_T_*x*_−MB shows an increased density of states in the vicinity of the Fermi level compared with bare Ti_3_C_2_T_*x*_ (Fig. 5g). This enhanced electronic coupling is consistent with the pronounced MB baseline current and the low charge‑transfer resistance (R_ct_) observed experimentally for the ICR‑MB‑MX‑11 laminate [56]. As shown in Fig. S12, the HOMO‑LUMO gap of isolated MB is 3.475 eV (spin‑up) and 3.048 eV (spin‑down), with electron density transfer from sulfur, nitrogen, and C = C bonds to C − C single bonds, highlighting a favorable charge‑transfer character that facilitates feasible electronic interaction with the MXene surface [19, 57].

In contrast, ERD adsorption yields a slightly more favorable binding energy of − 0.671 eV (Fig. 5d), with the aromatic quinazolinone core positioned at a somewhat greater distance from the basal plane (~ 3.1 Å; Fig. 5c). The charge-density-difference map is more localized around the ERD framework and adjacent surface sites (Fig. 5f), while the total DOS analysis shows a redistribution of states (Fig. 5h). ERD introduces additional unoccupied states above the Fermi level, and the Ti-centered DOS at E_F_ is reduced compared to the MB–MXene complex (Fig. 5g), indicating a reorganization of Ti-centered electronic states due to ERD and its stronger perturbation of the surface. This perturbation affects the electronic structure of ERD, as shown in Fig. S13, where it exhibits a narrower HOMO-LUMO gap (ΔE = 3.238 eV), indicating higher electronic reactivity and localized charge redistribution upon adsorption [58]. Evidence of interfacial electronic modulation is further supported by the projected density of states (DOS) for elements in individual hybrid systems (Fig. S14). The DOS for C, Ti, N, S, O, and H indicates differential hybridization of ERD and MB orbitals with Ti_3_C_2_T_*x*_. The MB-MXene system shows stronger Ti and S/N coupling near the Fermi level, consistent with delocalized charge transfer and higher conductivity [59, 60]. Conversely, ERD-MXene exhibits suppressed Ti states near EF and increased contributions from nitrogen and carbon in the unoccupied region, reflecting greater state localization and higher charge-transfer resistance [55]. This redistribution aligns with the experimentally observed increase in R_ct_ and the concentration-dependent suppression of the MB-DPV peak. The optimized geometries and electron-density mappings of MB and ERD are shown in Figs. 4(e-f) for reference.

**Fig. 6:**
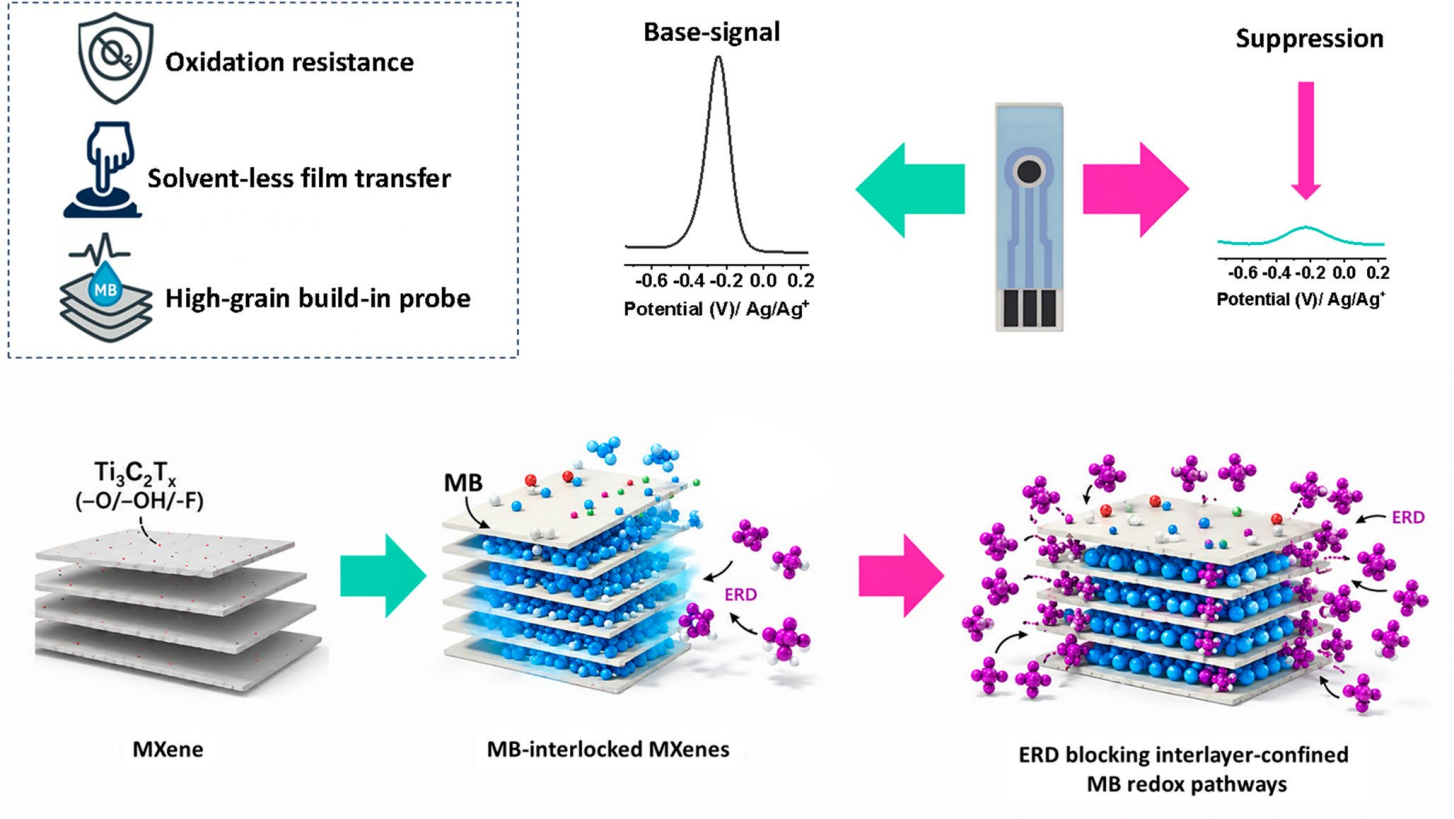
Schematic illustration of the ICR-MXene sensing concept, where MB confined within Ti_3_C_2_T_*x*_ galleries provides a built-in baseline signal on SPEs. ERD interacts near accessible gallery/interfacial regions, blocking or perturbing the confined MB redox pathway and suppressing the DPV peak, enabling reagent-free detection

Electrochemically, this correlates with the concentration-dependent suppression of the MB DPV signal (Fig. 6, right panel), enabling sensitive, direct quantification of ERD via non-covalent interfacial interactions. The sensing response is attributed to ERD-induced blocking and perturbation of the interlayer-confined MB redox network. MB is strongly stabilized within the negatively terminated Ti_3_C_2_T_*x*_ galleries through electrostatic and π–π interactions, forming a solid-state redox network. Upon ERD exposure, ERD molecules interact with accessible gallery entrances and the π-rich MXene/MB interface through non-covalent interactions, including π–π stacking, hydrogen bonding, and hydrophobic association. These interactions partially restrict ion/electron transport pathways to the confined MB sites and perturb the local electronic environment, thereby attenuating the MB redox peak.

## Conclusion

In summary, an interlayer-confined redox (ICR) MXene laminate is developed as a new solid-state platform for reagent-free electrochemical sensing. This architecture confines methylene blue (MB) within Ti_3_C_2_T_*x*_ galleries, forming a built-in redox network inside a freestanding laminate that can be directly transferred onto commercial SPEs. This concept integrates mediator confinement, laminate transferability, and reagent-free detection into a single sensing architecture, thereby avoiding the use of soluble mediators, biological recognition elements, and post-assembly surface functionalization commonly employed in conventional platforms. Structural and spectroscopic analyses confirmed that MB intercalation expands the MXene galleries while preserving the lamellar framework, in-plane crystallinity, and carbide structure without inducing crystalline TiO_2_ formation. Electrochemical optimization identified ICR-MB-MX-11 as the optimal composition, providing a stable MB redox signal at approximately − 0.25 V vs. the on-chip Ag pseudo-reference, low mediator leaching, good reproducibility, and an effective balance between redox activity and transport accessibility. Under optimized DPV conditions, the ICR-MB-MX-11 sensor detected ERD over 0.05–10.5 µM with a 0.01 µM LOD, negligible interference from common ions, metabolites, co-medications, structural analogs, and fouling agents, and recoveries of approximately 98–102% in spiked human urine. Compared with reported chromatographic, fluorescence, and electrochemical ERD assays, this platform offers a competitive working range, sensitivity, disposable SPE compatibility, and operation without added soluble mediators or complex surface chemistry. The sensing response is assigned to blocking-mediated inhibition of the interlayer-confined MB redox network. ERD interacts with accessible gallery and interfacial regions, leading to suppression of MB redox accessibility, supported by EIS and DFT evidence of increased charge-transfer resistance and electronic-state redistribution.

Overall, the adopted ICR strategy provides a new, yet simple and practical, route to constructing solid-state MXene sensors with mediator retention, low reagent consumption, and compatibility with portable electrochemical analysis. This concept highlights the practical value of solid-state electrochemical platforms in which the redox probe is pre-integrated into the sensing layer rather than added externally during measurement. This design eliminates the need for additional soluble probes, reduces user-handling steps, minimizes reagent consumption, and improves operational stability by retaining the mediator within the electrode structure. Such characteristics may also be advantageous for decentralized monitoring of chemically complex waters encountered in industrial, metallurgical, and resource-processing environments, where portable, low-reagent sensing platforms are increasingly needed. By tuning the MXene chemistry, confined mediator, and interfacial affinity toward selected targets, this solid-state platform could be adapted for other pharmaceuticals, environmental contaminants, and clinically relevant small molecules in complex samples. 

## Supplementary Information

Below is the link to the electronic supplementary material.

Supplementary Material 1

## Data Availability

No datasets were generated or analysed during the current study.
