# Supplementary material for: Interlayer-confined redox in Ti₃C₂Tₓ-MXene laminates: a reagent-free screen-printed platform for the detection of erdafitinib
Source: Mikrochim Acta. 2026 Jun 4;193(7):440. doi: 10.1007/s00604-026-08175-4 (PMC13236810; doi:10.1007/s00604-026-08175-4)
Supplement: Supplementary file 1 — Supplementary Material 1 [file 604_2026_8175_MOESM1_ESM.docx]

**Supporting Information**

**Interlayer-Confined Redox in Ti₃C₂Tₓ-MXene Laminates: A Reagent-Free Screen-Printed Platform for the Detection of Erdafitinib**

# 1 Materials and Methods

# 1.1 Synthesis of Ti_3_C_2_T*_x_* dispersion

MXene-Ti_3_C_2_T*_x_* nanosheets were synthesized by selectively etching aluminum from the MAX phase precursor [1, 2]. A hydrochloric acid (HCl) solution was prepared by diluting concentrated HCl (37% w/w, Sigma Aldrich) with deionized (DI) water. To ensure safety, care was taken when dissolving 3 grams of LiF into the HCl solution, as this process generates hydrofluoric acid (HF). Gradually, 3 grams of Ti_3_AlC_2_ MAX powder were added to the HCl + LiF solution to prevent overheating. The mixture was stirred and maintained at 40 °C for 24 h. After the etching process, several washing cycles were performed until the pH of the mixture reached 6 or higher. The resulting dispersion of Ti_3_C_2_T*_x_* nanosheets was stored in a dark bottle and used directly for film formation via vacuum filtration.

# 1.2 Computational details

Molecular-level DFT calculations for methylene blue (MB) and erdafitinib (ERD) were performed using the Gaussian 16 software package to analyze their stable geometric configurations of MB and ERD molecules [3]. The hybrid functional B3LYP and the 6-311G(2d,p) basis set were employed to obtain the minimum energy geometries in the gaseous state. The convergence criteria for both energy and forces were set to 10⁻⁶ Hartree throughout the optimization process. To accurately account for dispersion interactions, the Grimme’s DFT-D3 van der Waals correction was applied, ensuring more reliable optimization of the structures and more accurate calculations of their properties. Single point energy (SPE) calculations were performed on the optimized structures to calculate Gibbs free energies, Frontier Molecular Orbitals (FMOs), and Electrostatic Potential (ESP) densities using Gaussian 16. These calculations were designed to analyze the electronic properties of the molecules and examine the reactivity of different functional groups within them. Visualization of ESP surfaces, FMOs, and molecular geometries was performed using GaussView 6.0.16 software [4]. For the adsorption study of MB and ERD on the MXene surface, the 3D surface of MXene and the adsorbed complexes were optimized using the VASP software package. The PBE functional and a 350 eV cutoff energy were used for these calculations. After performing the SPE calculations, the Density of States (DOS) and electron density differences were computed using the Vaspkit tool. Visualization of the optimized 3D periodic surfaces and their complexes was done using VESTA software [5, 6].

# 1.3 Collection of urine samples

Fresh urine specimens (≈ approximately 50 mL) were collected from six healthy, drug-free adult volunteers from our laboratory (three men and three women, aged 22–35 years) with informed consent and anonymized before analysis. No clinical intervention, diagnosis, or personally identifiable information was involved. Each sample was centrifuged at 4,000 × g for 5 min to remove cellular debris, and the clear supernatant was passed through a sterile 0.22 µm PTFE syringe filter to eliminate residual particulates and microorganisms. The filtrate pH was adjusted to 6.0 ± 0.1 with dilute HCl/NaOH, then diluted 1: 1 (v/v) with 0.01 M phosphate-buffered saline (PBS, pH 6.0) to moderate matrix effects while maintaining the analyte within the sensor’s linear range. A 1 mM Erdafitinib (ERD) stock solution was freshly prepared in PBS (pH 6.0) and serially diluted to create 1 mL aliquots of the treated urine at seven concentrations spanning 0.05–10.5 µM, with each level prepared in triplicate. After vortex mixing for 30 s and a 10 min equilibration, a 10 µL sample was dropped on the ICR-MB-MX-11 electrode, and differential-pulse voltammograms were recorded with the fabricated ERD sensor under the same instrumental parameters used for calibration. Measured concentrations, standard deviations (SD), recoveries, and relative standard deviations (RSD) are summarized in **Table S1**. All recoveries (98.1–102.4%) and RSD values (≤ 3.3%) complied with FDA/ICH criteria for accuracy (95–105%) and precision (≤ 5%), demonstrating that the sensor provides reliable quantification of ERD in complex urinary matrices without appreciable interference.

**Figure S1** (a) UV-Vis spectra for MB dye showing an increasing typical peak with rising concentration, and corresponding (b) calibration curve, and (c) graph illustrating the retained concentration of MB in MX-MB-*x* electrodes, obtained by measuring the concentration of the filtrate subsequent to standard calibration.

**Figure S2** (a) SEM image of a representative MAX phase (Ti_3_AlC_2_), and (b) TEM image of etched and delaminated few-layer MXenes (Ti_3_C_2_T*_x_*).

**Figure S3** SEM topological assessment of (a) drop-casted MXene film, and (b) ICR-MB-MX-11 laminate showing a dense, continuous network with significantly fewer surface defects.

**Figure S4** can-rate-dependent CVs of ICR-MB-MX-11 recorded in PBS (pH 6.0) from 3 to 100 mV s^-1^, with corresponding $i_{p}$versus $v$, $i_{p}$versus $v^{1/2}$, and log $i_{p}$–log $v$plots for evaluating the charge-storage kinetics of the interlayer-confined MB redox process.

**Figure S5** Scan-rate-dependent CVs of (a-c) pristine Ti_3_C_2_T*_x_*/SPE and (b-d) ICR-MB-MX-11 recorded in 5 mM Ru(NH₃)_6_^3+^/^2+^, with corresponding $i_{p}$versus $v^{1/2}$plots used to estimate the apparent electrochemically active surface area and outer-sphere electron-transfer kinetics.

**Figure S6** Optimization of DPV (a) pulse amplitude and (b) pulse width for robust MB-based redox activity.

**Figure S7** UV-vis spectroscopy of standard MB and the PBS electrolyte after DPV runs.

**Figure S8** DPV-based inhibition response optimization for ICR-MB-MC-11 electrode against ERD under different pH levels from 3 to 9.

**Figure S9** DPV-based intra-electrode reproducibility for ICR-MB-MX-11 electrode against ERD during 10 cycles.

**Figure S10** DPV curve for ICR-MB-MX-11 with representative ascorbic acid (AA) interferent showing negligible inhibition.

**Figure S11** Representative DPV curve for ICR-MB-MX-11 for the spiked samples, indicating stable signal response and minimal matrix-induced distortion.


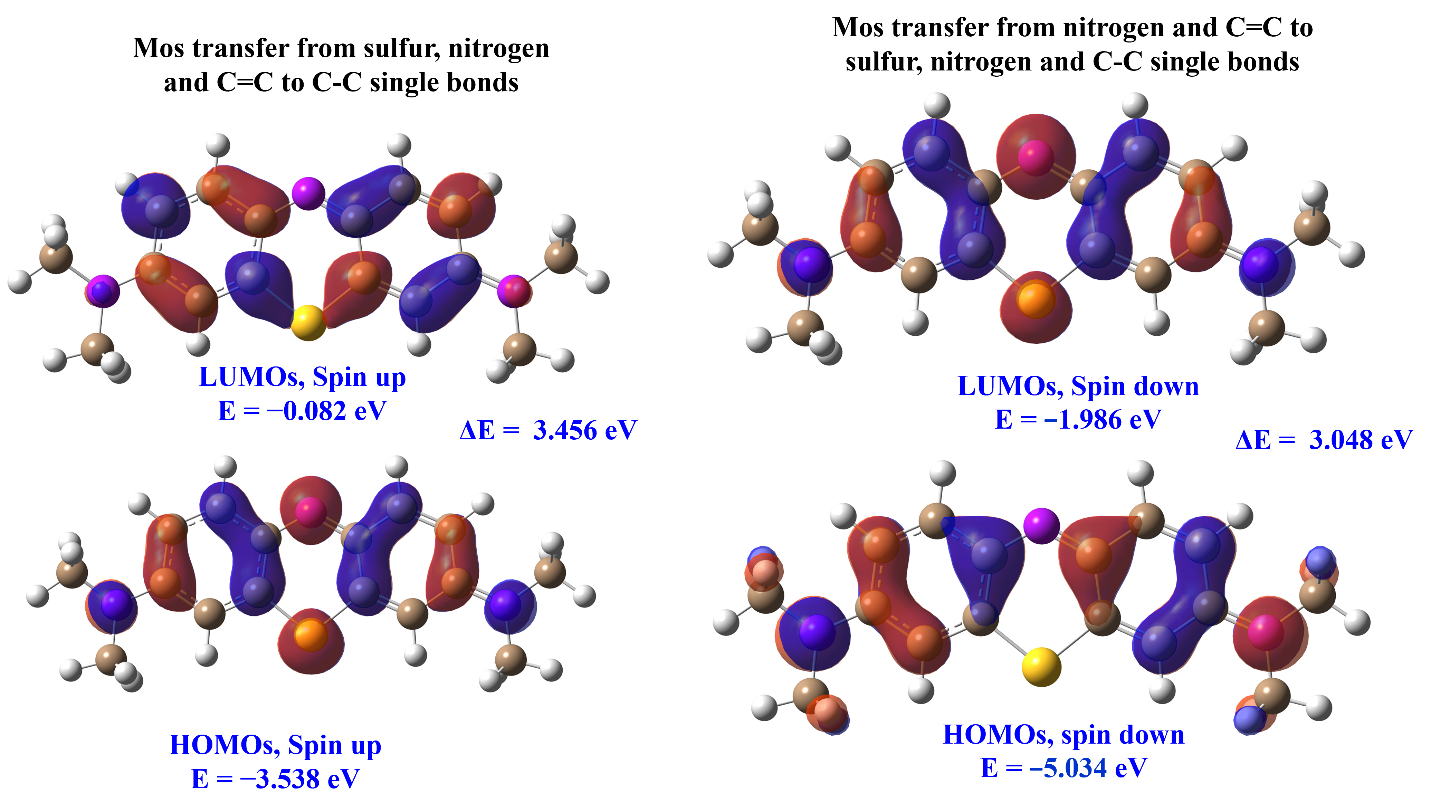


**Figure S12** Electron density transfer in methylene blue (MB) from sulfur, nitrogen, and C=C bonds to C–C single bonds, with HOMO-LUMO energies and gaps (ΔE: 3.475 eV for spin-up, 3.048 eV for spin-down), highlighting its redox activity.


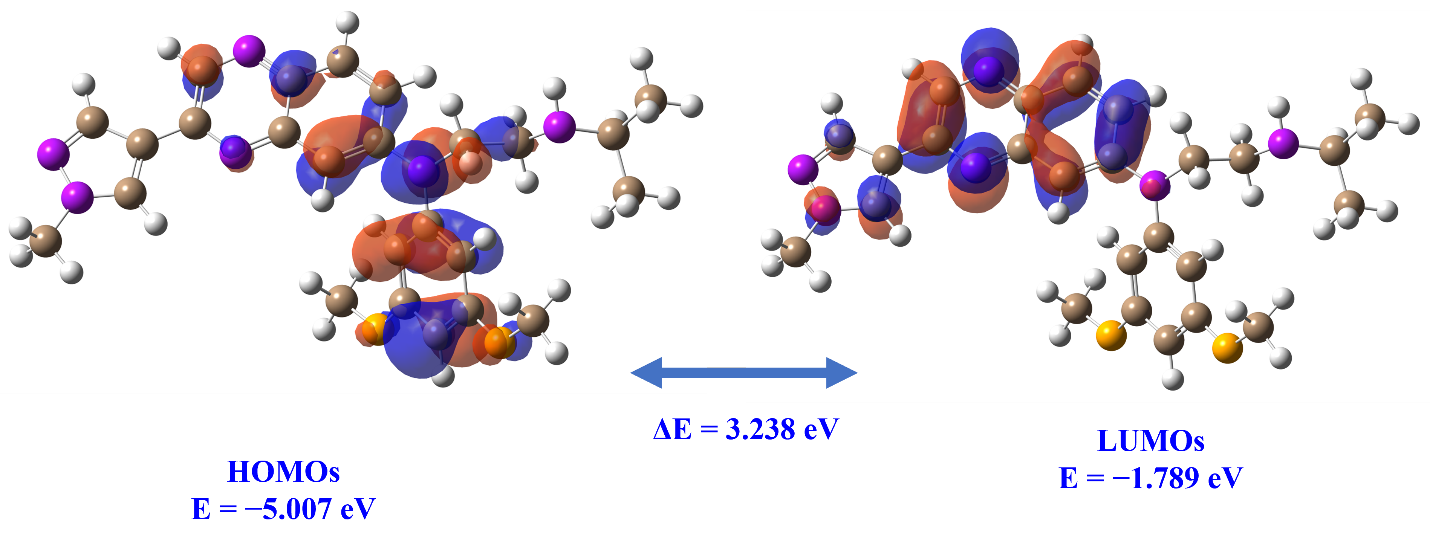


**Figure S13** HOMO-LUMO analysis of ERD showing the highest occupied molecular orbitals (HOMOs) at -0.184 Ha and lowest unoccupied molecular orbitals (LUMOs) at -0.065 Ha. The energy gap (ΔE = 0.119 Ha) reflects the electronic reactivity of ERD.

**Figure S14** DOS for various elements (C, Ti, N, S, O, H) in the MXene-ERD and MXene-MB systems.

**Table S1** Spike-and-recovery results for ERD in human urine samples detected using the developed sensor.

| **Sample** | **Spiked (µM)** | **Found (µM ± SD) *** | **Recovery (%)** | **RSD (%)** |
| --- | --- | --- | --- | --- |
| 1 | 0.05 | 0.051 ± 0.001 | 102.3 | 2.30 |
| 2 | 0.25 | 0.252 ± 0.005 | 101.0 | 1.86 |
| 3 | 0.50 | 0.491 ± 0.008 | 98.30 | 1.68 |
| 4 | 1.00 | 1.024 ± 0.034 | 102.4 | 3.29 |
| 5 | 2.50 | 2.507 ± 0.065 | 100.3 | 2.60 |
| 6 | 5.00 | 4.904 ± 0.114 | 98.10 | 2.32 |
| 7 | 8.50 | 8.602 ± 0.187 | 101.2 | 2.17 |

*Triplicate analyses for each level

**Table S2** Analytical comparison of ICR-MB-MX-11 with current sensors and detection techniques for ERD.

| **S. No** | **Electrode material** | **Measurement Technique** | **Peak Potential (V)** | **linear range (μM)** | **Limit of detection (μM)** | **References** |
| --- | --- | --- | --- | --- | --- | --- |
| 1 | \| Co/ZIF-12 porous carbon/GCE \|  \| \| --- \| --- \| | DPV | 0.75 | 0.01–7.38 | 0.18 | [7] |
| 2 | – | HPLC–UV | – | 0.11 – 4.50 |  | [8] |
| 3 | – | HPLC–UV | – | 1.10 – 1130 | 0.45 | [9] |
| 4 | – | Spectrofluorimetric | – | 0.11 – 1.80 | 0.33 | [10] |
| 5 | Gd_2_(WO_4_)_3_–P@rGO heterostructure/GCE | DPV | 0.85 | 0.00001–0.8 | 0.0000024  (2.4 pM) | [11] |
| 6 | ICR-MB-MX-11 | DPV | -0.25  (MB-probe) | 0.05–10.5 | 0.01 | ***This work*** |

Co/ZIF-12 porous carbon/GCE: glassy-carbon electrode coated with cobalt-doped ZIF–12–derived porous carbon; Gd_2_(WO_4_)_3_–P@rGO/GCE: glassy-carbon electrode modified with phosphorus-doped Gd_2_(WO_4_)_3_ and reduced graphene oxide heterostructure; ICR-MB-MX-11: screen-printed electrode modified with an interlayer-confined methylene blue/Ti_3_C_2_T*_x_* MXene laminate.

# References

1. Kumar, J., et al., *Robust Electrochemical Sensors for Detection of Isoprenaline Using Hexagonal Co3O4 Nanoplates Embedded in Few-Layer Ti3C2Tx Nanosheets.* ACS Applied Nano Materials, 2022. **5**(8): p. 11352-11360.

2. Li, K., et al., *Alkali-induced porous MXene/carbon nanotube-based film electrodes for supercapacitors.* ACS Applied Nano Materials, 2022. **5**(3): p. 4180-4186.

3. Frisch, M.J., et al., *Gaussian 16 Rev. C.01*. 2016: Wallingford, CT.

4. Roy Dennington, T.K., and John Millam, *GaussView, Version 6.1, Semichem Inc., Shawnee Mission, KS*. 2019.

5. Emamian, S., et al., *Exploring Nature and Predicting Strength of Hydrogen Bonds: A Correlation Analysis Between Atoms-in-Molecules Descriptors, Binding Energies, and Energy Components of Symmetry-Adapted Perturbation Theory.* J Comput Chem, 2019. **40**(32): p. 2868-2881.

6. Lu, T. and F. Chen, *Multiwfn: a multifunctional wavefunction analyzer.* J Comput Chem, 2012. **33**(5): p. 580-92.

7. Yildir, M.H., et al., *Pioneering electrochemical detection unveils erdafitinib: a breakthrough in anticancer agent determination.* Microchimica Acta, 2024. **191**(4): p. 221.

8. Elawady, T., et al., *HPLC-UV determination of erdafitinib in mouse plasma and its application to pharmacokinetic studies.* Journal of Chromatography B, 2021. **1171**: p. 122629.

9. Elawady, T., et al., *LC-MS/MS determination of erdafitinib in human plasma after SPE: Investigation of the method greenness.* Microchemical Journal, 2020. **154**: p. 104555.

10. Elawady, T., et al., *Utility of Kolliphor RH 40 in micellar sensitized fluorescence of the novel tyrosine kinase inhibitor “Erdafitinib”: application to human plasma.* Spectrochimica Acta Part A: Molecular and Biomolecular Spectroscopy, 2022. **278**: p. 121327.

11. Mohamed, R.M.K., et al., *Engineering a Gd₂(WO₄)₃–P@rGO heterostructure for enhanced electrochemical sensing and therapeutic drug monitoring of erdafitinib.* Microchimica Acta, 2026. **193**(3): p. 165.
